# Cervical spine sensorimotor deficits persist in people post-concussion despite minimal symptoms

**DOI:** 10.1080/07853890.2024.2422048

**Published:** 2024-11-07

**Authors:** Anthony E. Toby Kinney, Sierra L. K. Richmond, Ryan L. Mizner

**Affiliations:** a Physical Therapy Program, University of Colorado Anschutz Medical Campus, Aurora, CO, USA; b Mt. Ascension Physical Therapy, Helena, MT, USA; c School of Physical Therapy & Rehabilitation Science, University of Montana, Missoula, MT, USA

**Keywords:** Post-concussion, cervical spine, joint position error

## Abstract

**Background:**

The mechanisms of a concussion place stress on the cervical spine like that of a whiplash event, which can result in cervical spine dysfunction. This study aimed to determine if underlying cervical spine mobility and sensorimotor function deficits occur in individuals who are post-concussion with near resolution of symptoms.

**Methods:**

Twenty-five participants with a self-reported concussive event within a year (PC group: post-concussion 157 + 120 d, 9 men, age: 25 ± 8 yr) and 26 comparable peers (Peer group, 9 men, age: 25 ± 7 yr) were tested. The Post-Concussion Symptom Scale (PCSS) quantified residual concussion symptoms. Participants completed cervical joint position error (JPE) and cervical spine joint mobility tests blinded from each other. Group mean differences were analyzed using t-tests.

**Results:**

The PC group had minimal symptoms (PCSS = 6.8 ± 6.5) but substantial differences in JPE tests compared to the Peer group (PC = 7.4 ± 1.8 cm; PG = 5.6 ± 1.1 cm; *p* < .001). Those PC participants with pain during joint testing (*n* = 15) had worse JPE (Painful = 8.1 ± 1.8 cm, No-pain = 6.3 ± 1.6 cm; *p* = .02) and less averaged lower cervical spine joint mobility compared to PC participants without pain (Painful = 0.66 ± 0.22, No-pain = 0.87 ± 0.19; *p* = .02, Normal motion = 1.0).

**Conclusion:**

Following a concussion, it is a reasonable recommendation to screen the cervical spine to identify impairments in joint mobility and JPE that contribute to neck dysfunction.

## Introduction

Approximately one in four Americans will experience a concussion in their lifetime [1]. A concussion represents a form of mild traumatic brain injury (mTBI) because of transmitted forces to the head. Of those individuals who experience a concussion 80% will have neurocognitive signs and symptoms that resolve within three weeks of injury [2,3]. The biomechanical mechanisms involved in trauma to the head can propagate large forces to the cervical spine, similar to that of a whiplash mechanism. The forces involved in a concussive event can induce tremendous stresses to the cervical spine’s connective tissues (e.g. tension on joint capsules or muscles) that could create resultant injury to the neck. It is not surprising that a number of individuals who present with mild traumatic brain injury (mTBI) to the emergency department have been found to have acute neck pain [4]. Additionally, previous research has linked neck dysfunction to post-concussion symptoms [5–7]. A recent study by Oyekan et al. found that 47% of post-concussion patients reported neck pain and 22.2% reported difficult moving their neck [8]. Additionally, previous research has reported a connection with concomitant neck pain and protracted recovery from concussion [8–11]

Individuals with neck trauma, such as whiplash injury, often exhibit abnormal cervical spine joint mobility, impaired head repositioning, and impaired kinesthetic awareness [12,13]. Previous research has recognized sensorimotor changes occur in the cervical spine following a concussion [14–17]. Assessment of joint position error (JPE) of the cervical spine has been recommended as part of an evaluation following a concussion [18].

In addition to changes in JPE a high prevalence (85%) of moderate to severe pain with manual mobility testing of the upper cervical spine in patients seeking rehabilitation for post-concussive symptoms that last > 10 days has been previously reported [6]. While cervical symptoms in people engaging in rehabilitation may be expected, the prevalence of cervical spine symptoms of people outside of formal care, including rehabilitation, may be particularly informative as they represent a larger portion of all people with concussions. Work by Kennedy et al. [6] focused on the upper cervical spine, while understanding of the lower cervical spine segments remains unreported. Lower cervical spine dysfunction may contribute substantially to clinical exam items such as head repositioning and neck pain. Lastly, the presence of an associative link between cervical spine mobility and kinesthetic awareness is an important missing element that may provide a deeper understanding of persistent post-concussive symptoms. A more complete understanding of the extent of post-concussion cervicogenic impairments would inform the need for future cervical spine screening and spine-related treatment.

Developing a deeper understanding of the clinical presentation of people with concussion that may identify unique and lasting physical impairments is warranted. The purpose of the study was to explore the presence of underlying impairments of the cervical spine in joint mobility (painful and restricted mobility) and kinesthetic awareness using joint position error in individuals tested within a year of a concussive event. Given the traumatic nature of the injury, we hypothesized that patients with a prior concussion would exhibit restricted cervical spine mobility with manual examination, and have impaired kinesthetic awareness compared to age-matched peers with no recent concussion. Further, we expected these impairments would linger and be present despite the near resolution of neurocognitive signs and symptoms.

## Methods

This study involved measuring a group of people who self-reported having a concussion within a year compared to a similarly aged peer group who denied having a concussion in the past two year. Both groups were tested in the School of Physical Therapy and Rehabilitation Science at the University of Montana.

### Participants

A purposive sampling method was employed to recruit all participants. Volunteers were adults who could read and understand English, aged 18–50 years old who believed they had a concussion in the past year, and were recruited using word of mouth, paper flyers, and email advertisements. For the post-concussion cohort, 35 potential participants expressed interest in the study and were screened over the phone for eligibility. Inclusion criteria for the test group included a history of concussion within 12 months. The presence of a concussion was based on answering ‘yes’ to at least 1 of our four screening criteria (see Table 1) from the Consensus Statement on Concussion in Sport, 2012 [19]. Exclusion criteria included a history of current or past vestibular dysfunction or balance condition, inflammatory disease, known neurological condition, connective tissue disease, lower extremity surgery or fracture in the past 6 months, systemic illness, history of 2 or more falls in the past 6 months, malignancy or neoplasm, history of intracranial bleed, osteoporosis, history of a neck fracture, pregnancy, cervical spine surgery, psychogenic disorders, radiating extremity pain, dizziness or vertigo, drug abuse, infection, and cervical/vertebral artery dysfunction. If participants were deemed eligible for the study, then a testing day and time was arranged. In total, 25 participants (16 women) who met the definition of a concussion or who had a medically diagnosed concussion (post-concussion) and/or met the definition of a concussive event were recruited. See Table 2 for the demographics of the post-concussion group.

**Table 1.** Questions asked to participants to determine concussed event.

(1) Did you have a direct blow to the head, face, neck or or elsewhere on the body with an ‘impulsive’ force transmitted to the head?
(2) Did you have a rapid onset of short-lived impairment of neurologic function that resolved spontaneously?
(3) Did you feel like you had functional disturbance to your head/brain?
(4) Did you feel like you had clinical symptoms that may or may not have involved a loss of consciousness?

Questions based on definition of concussion from Consensus Statement on concussion in Sport, 2012 (McCrory et al. [19]).

**Table 2.** Demographics of test groups.

	Post-Concussed Group (*n* = 25)	Peer Group (*n* = 26)
**Sex (Men/Women)**	9/16	8/18
**Age (years)**	25 + 8 (18–45)	25 + 7 (18–52)
**Time Since Concussion (days)**	157 + 120	n/a
**% of subjects who met definition of concussion**	Criteria 1: 100% (25/25) Criteria 2: 92% (23/25) Criteria 3: 96% (24/25) Criteria 4: 76% (19/25)	n/a

n: number; S.D.: standard deviation; Mean ± S.D (range).

Recruitment and data collection for a peer group took place separately as part of a previous investigation. Exclusion criteria were if the subject had a known vestibular and/or balance condition, known neurological condition, lower extremity surgery or fracture in the past six months, and/or a history of two or more falls in the past six months. We had 26 subjects who volunteered for the study. Participants in the peer group were queried about previous neck pain and concussion. Eight of the 26 peer group participants had previous neck pain in their lifetime and nine reported having a possible concussion in their lifetime but denied having a possible concussion in the previous two years. At the time of testing, subjects reported not having neck pain nor having a concussion in at least 2 years.

### Data collection

Prior to testing, participants received instruction in the sequence of data collection by the primary author and provided informed consent. Participants did not have reports of neck pain or discomfort. Participants completed a Post-Concussion Symptom Scale (PCSS), a 22-item scale used to quantify the severity of symptoms following a concussion [20]. Symptoms are rated and scored on a 7 point 0–6 ordinal scale where a score of ‘0’ is the equivalent of no symptoms, while a ‘6’ is considered severe symptoms. The total possible points are 132 and the lowest score is zero. The PCSS is a self-report scale commonly used following concussion and has good psychometric properties [21]. In our study a symptom score of 50 or more was used as an artificial cut off due to the concern of overall symptom severity and irritability. One subject screened had a score >50 on the day of testing and was excluded from the study. The individual was instructed to follow-up with their respective medical provider as symptoms had increased since the screening process.

**Figure 1.**
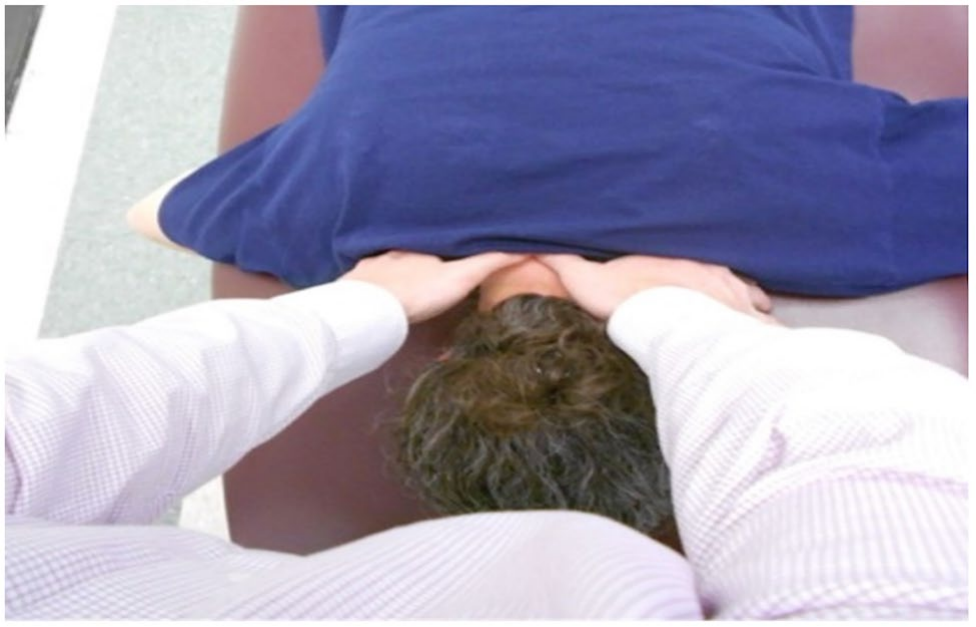
Cervical spine segmental mobility assessment (Maitland [24]). The individuals in this photo have provided written consent to use this image in the published manuscript.

*Cervical spine segmental joint mobility* was completed on the PC group following signed informed consent and completion of the Post-Concussion Symptom Scale. Although reliability and agreement of segmental testing have reported variability it is a commonly used clinical assessment technique [22,23]. Segmental spine joint mobility (Figure 1) was performed as described by Maitland [24] by a manual therapy fellowship-trained primary clinician investigator in a separate room to maintain assessor blinding of JPE test results. Joint mobility was graded on a 3-point scale for each level as either ‘hypermobile,’ ‘normal,’ or ‘hypomobile.’ Each level underwent a central PA, and respective unilateral PAs (left and right) as indicated. Pain with testing was recorded for each of the three tests for every segment using an 11-point Likert pain scale

**Figure 2.**
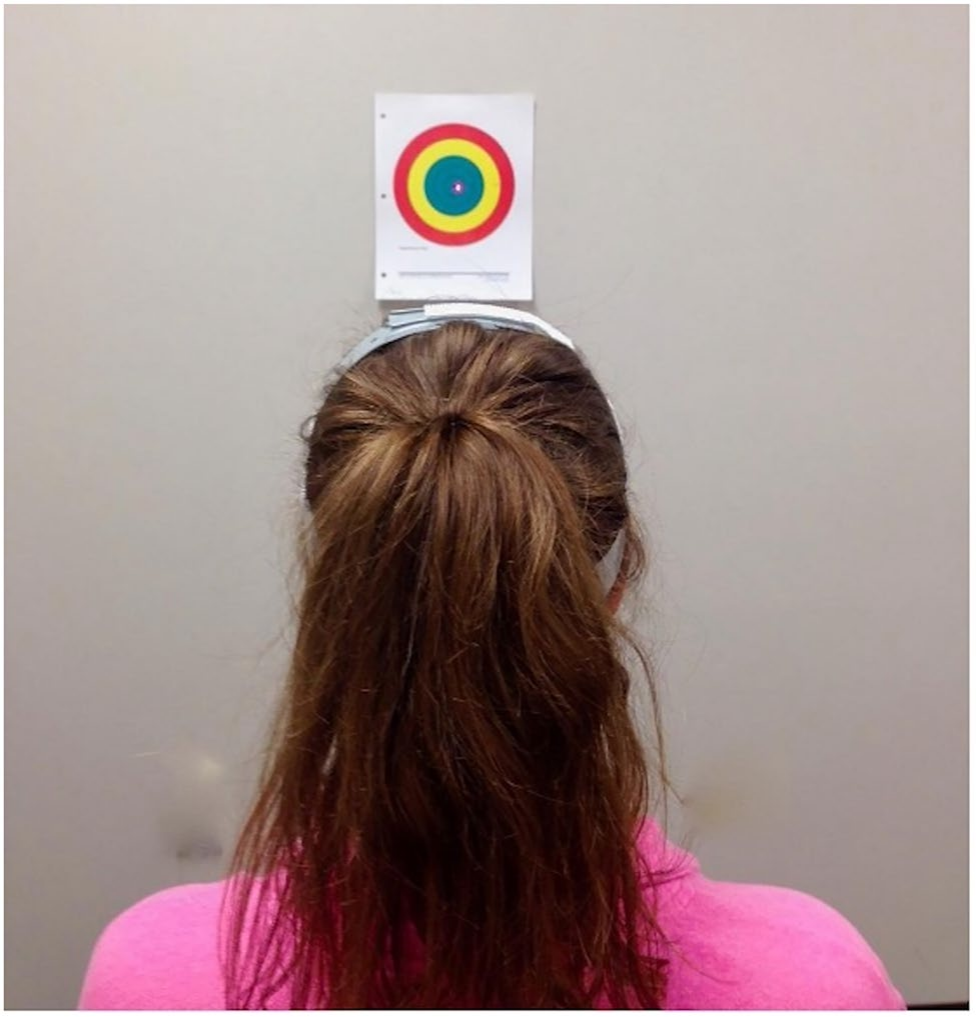
Cervical joint position error (JPE) set-up (based on Revel [25] and pre-formatted target from Landel (https://skillworks.biz/wp-content/uploads/2023/11/JPE-Target-Accurate-Size-Color.pdf)). The individual in this photo has provided written consent to use this image in the published manuscript.

*Cervical joint position error (JPE)* tests were completed with eyes closed and with a head-mounted laser quantifying motion as described by Revel [25]. A target bullseye created by Landel (https://skillworks.biz/calibrated-jpe-target-more-accurate-than-ever/) based on work by Treleaven et al. [13] was used (See Figure 2). This test technique has accurately identified residual performance deficits in persons with lasting and chronic neck pain [25] or recent neck trauma [13,26] compared to healthy injured peers. Prior studies report high inter-rater and intra-rater reliability with patients with whiplash (ICC = 0.97 and 0.98 respectively) [27]. A written script was used to ensure consistent instruction across participants. All participants sat with their feet flat on the floor with instructions to keep their body still and facing forward. The subjects started with the laser centered and in-line with the bullseye of a paper target spaced 0.9 m from the patient. Participants were instructed to move through their pain free range using only their head and neck with eyes closed before returning to their perceived starting position. They verbally reported when they felt they returned to the initial starting position. Data for each JPE movement was quantified as the distance from the starting target to the end-test laser beam position in cm on a target. For the sake of clinical pragmatism, time and capturing planes of motion ten neck test movements were completed. The sequence of testing was performed in the following order: left rotation to center, right rotation to center, flexion to center, extension to center, the horizontal figure of 8 pattern in the participant’s preferred direction of right to left, left to right to center, right to left to center, flexion to extension to center, extension to flexion to center, and ending with a second horizontal figure of 8 completed in the opposite direction of the first figure of 8. In each instance the patient was instructed to reposition to their natural resting posture. The distances were averaged across all movements for an averaged JPE distance. A seven cm distance from the bullseye of the target is equivalent to the 4.5 degree allowance used to discern normal performance [25].

### Data management and statistical analysis

An a priori sample size was determined using G*power 3.1.9.2 [28,29]. We anticipated a large effect size of 0.8 in the joint position testing data. A sample size of 26 in a group would be needed to adequately test this difference using a two-tailed independent t-test with a beta level of 0.8 and an alpha level of 0.05. Descriptive statistics of mean, standard deviation, and ranges were used to describe patient demographics and variables of interest. JPE data movements were tested with a Skewness statistic and any movement with a value of 2.0 were searched for outliers (z score >2.58). Outliers in these movements were recorded to a value of 1 unit greater than the next most extreme value [30,31]. After recording the outliers all data could be considered normally distributed. Sex distribution between groups was tested using a Chi-squared test. Independent samples t-tests were used to identify group or category differences in age, JPE, pain with segmental mobility testing, and relative motion with spine joint mobility tests. All tests between groups for JPE were one-tailed with the assumption that the PC group would have greater error. Levene’s test for equality of variance was completed and the p-value of the t-test for equality of means was adjusted in those areas of unequal variance. All tests were completed using SPSS (IBM Corp [32]) Alpha level for statistical significance was set at 0.05. The cervical spine was also divided into an upper (all tests on C1 and C2) and lower (all tests from C3 to C7) designation given the unique structure and increased segmental mobility possible in the upper segments.

## Results

Following initial screening, 26 post-concussion group participants met the inclusion criteria for testing (See Figure 2), 25 completed testing, and 26 peer group participants also completed testing. Participant demographic information is summarized in Table 2. There were no significant differences in groups for age (*p* = .986) or sex distributions (*p* = .692).

### Symptom presentation

The post-concussion group had minimal symptoms on the Post-Concussion Symptom Scale (PCSS mean ± standard deviation = 6.8 ± 6.5, range:0-17) on the day of testing. A summary of collective PCSS responses per symptom from the post-concussed group can be found in Table 3. Fatigue (48%), drowsiness (40%), and difficulty concentrating (36%) were the most common reported symptom from the PCSS.

**Table 3.** Detailed breakdown of PCSS reports and scores for post-concussed individuals.

PCSS Symptom	% reporting symptom	Mean	Std Dev.	Score of ‘0’ Count	Score of ‘1’ Count	Score of ‘2’ Count	Score of ‘3’ Count	Score of ‘4’ Count
Fatigue	48%	0.60	0.71	13	9	3	0	0
Drowsiness	40%	0.64	0.81	13	9	2	1	0
Difficulty concentrating	36%	0.64	0.81	14	6	5	0	0
Headache	32%	0.52	0.87	17	4	3	1	0
Trouble Falling Asleep	32%	0.52	0.82	16	6	2	1	0
Loss of Sleep	32%	0.60	0.96	16	5	2	2	0
Difficulty remembering	32%	0.52	0.77	16	5	4	0	0
Irritability	24%	0.44	0.87	17	7	0	0	1
Feeling ‘slow’	24%	0.32	0.56	18	6	1	0	0
Light Sensitivity	20%	0.40	0.76	18	5	1	1	0
Feeling ‘foggy’	20%	0.24	0.52	20	4	1	0	0
Dizziness	16%	0.20	0.50	21	3	1	0	0
Excessive Sleep	16%	0.24	0.60	21	2	2	0	0
Sadness	12%	0.16	0.37	21	4	0	0	0
Visual problems	12%	0.12	0.33	22	3	0	0	0
Balance problems	8%	0.20	0.65	22	2	0	1	0
Noise Sensitivity	8%	0.16	0.47	22	2	1	0	0
More emotional	8%	0.08	0.28	23	2	0	0	0
Nausea	8%	0.04	0.20	24	1	0	0	0
Numbness	4%	0.08	0.40	24	0	1	0	0
Nervousness	0%	0.04	0.20	24	1	0	0	0
Vomiting	0%	0.00	0.00	25	0	0	0	0

PCSS: Post-Concussion Symptom Scale.

### Joint position error

**Figure 3.**
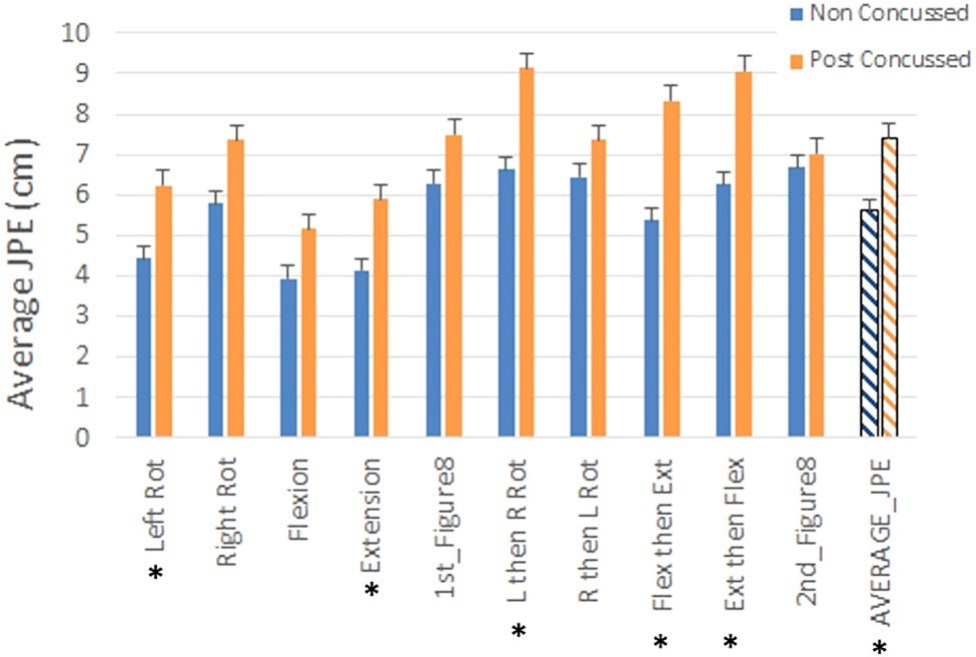
Comparison of joint position error (JPE) in post-concussed and peer (“non-concussed”) groups. Data presented is average and standard error bars. All ten movements and the averaged JPE across all movements is included. *p < .05 comparison between groups using independent t-tests. Ext: Extension; Figure8: horizontal figure 8 pattern; Flex: flexion; JPE: Joint Position Error; L: left; R: right; Rot: rotation.

In Figure 3, a comparison of joint position error (JPE) in post-concussed (PC) to peer groups can be found. The extension to center movement had a skewness statistic of 2.2 and two outliers in the PC group were recoded as described in the method section before analysis. Averaged JPE for each of the ten movements and an average across the ten movements was performed. The PC group had greater distance for all JPE movements, but they only reached statistical significance for left rotation (PC = 6.2 ± 3.8 cm; PG = 4.4 ± 2.5 cm; *p* = .025), extension (PC = 5.9 ± 4.6 cm; PG = 4.1 ± 2.5 cm; *p* = .030), left then right rotation (PC = 9.1 ± 5.0 cm; PG = 6.6 ± 3.7 cm; *p* = .004), flexion then extension (PC = 8.3 ± 4.3 cm; PG = 5.4 ± 3.4 cm; *p* = .004), and extension then flexion (PC = 9.1 ± 4.7 cm; PG = 6.3 ± 3.5 cm; *p* = .009). The post-concussed group demonstrated a greater average distance from the final target across all the tests used during the JPE tests compared to the peer (PG) group (PC = 7.4 ± 1.8 cm; PG = 5.6 ± 1.1 cm; *p* < .001).

### Joint mobility testing

**Figure 4.**
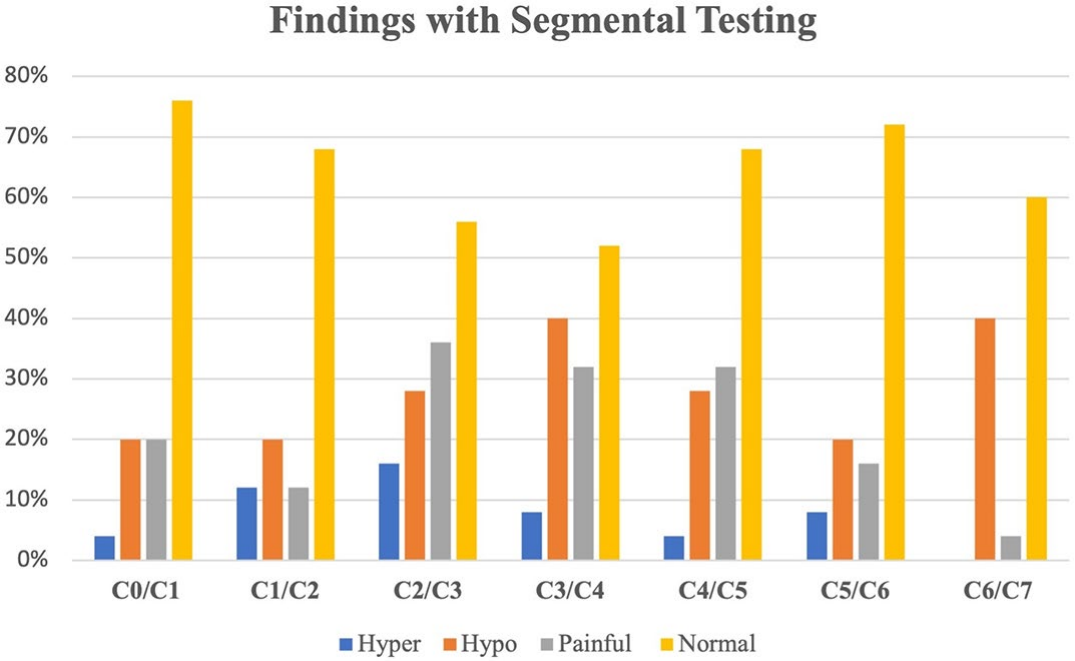
Frequency of segmental findings of the cervical spine in the post-concussed (PC) group.

Segmental findings of the cervical spine in the post-concussed (PC) group can be found in Figure 4. Participants in the PC group with pain during joint mobility testing (*n* = 15) had worse JPE (Painful = 8.1 ± 1.8 cm, No-pain = 6.3 ± 1.6 cm; *p* = .02) and reduced average lower cervical spine joint mobility compared to participants without pain (Painful = 0.66 ± 0.22, No-pain = 0.87 ± 0.19; *p* = .02, Normal motion = 1.0).

## Discussion

This cross-sectional, single-session study demonstrates the presence of underlying impaired cervical spine joint segmental mobility and kinesthetic awareness in individuals tested within a year of a defined concussive event. Similar to people with neck trauma, such as whiplash [33], people tested within a year of concussion have greater impairments in JPE than matched peers. In the PC group 60% (15/25) of participants had at least one painful segment with mobility testing of their cervical spine. Those subjects who had pain with mobility testing also had worse JPE and restricted segmental mobility in their lower cervical spine (C3–C7) compared to people with concussion that did not have pain with segmental motion testing. It is worth noting that neck pain was found with segmental assessment despite participants denying neck pain with screening question.

The findings from this study support the notion that joint position error testing should be considered as part of cervical spine screening following concussion even if individuals are asymptomatic. The difference in averaged JPE across movements was large (effect size > 1) and the average JPE fell outside the normal range of error of 7 cm as described by Revel [25] whereas our peer group was well below that threshold with an average value of 5.6 cm. Many of the JPE movements performed were significantly different between cohorts and 7 of the 11 movements had an average that fell outside the 7 cm cut point of normal, whereas none of the movements had error that fell beyond the normal range. Also, we found a similar pattern to previously reported findings in whiplash in that JPE worsened in the presence of painful neck symptoms [34]. In our study, participants denied having neck pain, but indicated a painful segmental with cervical mobility testing. Cervical spine proprioception relies on mechanoreceptor input from the musculature and articular components of the cervical spine to detect changes in the angles of the joints [35,36]. The current study describes meaningful alterations in JPE that coincides and is associated with cervical spine symptoms and impairments.

A recent study of male rugby players by Farley et al. found an association of poor cervical joint position error testing and the incidence of concussion, especially in the instance of error in the right rotation joint position error [37]. While our study did not include baseline joint position error testing it may indicate that there is a likely impact on cervical sensorimotor function. Although our study did not focus on studying rugby players, similarly, our study noted deficits in right rotation deficits and left-to-right rotation, but in post-concussed individuals. Further research should explore baseline testing and post-concussion testing to further explore if cervical joint position error is a modifiable risk factor.

Our work adds to prior case series work by Kennedy et al. who studied post-concussed individuals who were seeking treatment care at a multidisciplinary concussion treatment service due to their post-concussion symptoms [6]. The authors found that 90% of those concussed had persistent neck problems, with 85% of those reporting moderate to severe pain on occiput to C3-4 segmental manual palpatory assessment. In contrast, participants in our study were not actively seeking out care or treatment and they were tested much later in recovery (7.5 vs. 22.4 weeks from concussion respectively). It is important to note that while our participants did have symptoms based on the PCSS, the symptoms were minimal. In fact, previous research has found a similar pattern and symptom intensity of post-concussion type symptoms in non-concussed healthy individuals [38–41].

Still, 60% of our cohort reported some pain with segmental motion testing. Cervical spine examination and screening by a trained health care professional would reveal important impairments that are otherwise unnoticed by interviews or surveys. This finding is consistent with the work of Galea, O’Leary & Treleaven, who found that individuals 4 weeks to 6 months post mild Traumatic Brain Injury (mTBI) who did not have symptoms from self-report or surveys, yet still had cervical dysfunction [15,16]. We discovered that pain with joint mobility testing was a good indicator for a higher likelihood for impaired kinesthesia, as patients with pain with segmental testing also had abnormally high head repositioning errors. It is conceivable that for many patients a substantial degree of cervical spine dysfunction could go unnoticed during daily tasks but would be clearly evident with the clinical exam elements included in the current study. Early detection and intervention of impairments of the neck may diminish the possibility of developing chronic neck pain.

The inclusion of segmental mobility testing of lower cervical motion segments is another novel addition from prior studies [6]. The segments of the cervical spine work in conjunction to deliver overall neck motion with the upper cervical spine segements contributing to the greatest proportion of available neck motion [42,43]. Given the strong relationships and regional interdependency of the upper and lower cervical spine during head motion it is imperative to have optimal motion mechanics. In our study there was an association with cervical spine segmental dysfunction and greater joint position error, especially related to hypomobility in the lower cervical spine segments. Restricted lower cervical motion may prevent optimal shared motion of the cervical spine resulting in pathoanatomical conditions of the cervical spine and subsequent neck pain. The results of this study provide support for screening the cervical spine, especially joint-position error and cervical segmental mobility. As previous research as indicated, pain and dysfunction of the cervical spine results in prolonged recovery from concussion [8–11]. Therefore, early assessment of musculoskeletal impairments of the cervical spine and targeted intervention could minimize prolonged neck dysfunction and protracted recovery from concussion. One type of intervention, manual therapy, to the cervical spine could be beneficial to improve neck mobility and normalize function [44–47]. Future research could pursue the premise that restoring normal motion through manual and self-mobilization techniques could ease symptoms in other regional painful motion segments. In particular it could help reduce painful response with mobility testing, but it also may have some impact on common concussive symptoms such as dizziness or balance disruption.

Completing regular cervical spine screening exams for people with concussion could be particularly relevant as there is evidence that patients with cervical spine dysfunction could benefit from an intervention that includes manual therapy [48]. Previous research has demonstrated that interventions focused on the rehabilitation of cervical kinesthetic awareness have resulted in improvements in neck joint position error [12,49]. Palmgren et al. found that over a five-week period, chiropractic care in the form of high-velocity low-amplitude manipulation, proprioceptive neuromuscular facilitation, trigger point release, and spinal exercises improved head repositioning accuracy and decreased symptoms in patients with chronic neck pain [50]. Gong found that joint manipulation and massage directed to the cervical spine improved joint position sense and reduction in joint position error compared to healthy adult participants [51]. Therefore, it would be logical that cervical joint mobility may play a role in cervical joint position error. By better understanding the neuromusculoskeletal impairments seen in individuals who are without neurocognitive signs and symptoms following a concussion, clinicians may better direct interventions to prevent possible dysfunction of the cervical spine. Our work suggests manual therapy that targets painful cervical spine segments may help reduce JPE and an intervention that address reduced lower spine mobility may have some influence on pain symptoms in the spine.

## Limitations

The current study represents a progressive step forward in the existing evidence, but the cross sectional, single session design does have some important limitations. We recognize that some of the peer group reported previous neck pain and concussion in their lifetime more than 2 years prior to testing. Thus, there is some risk we are underestimating the group differences in JPE due to the potential for some residual impairment in the peer group from long-standing injuries. It is challenging to find a healthy comparison population without concussion and neck pain because of the prevalence of concussion (28.9%) and neck pain (mean of 23.1%) in the general population [1,52]. Participants were not tested in the acute injury stage; therefore, the early presentation of cervical spine dysfunctions remains unknown. Additionally, we relied on participants self-reporting of events and symptoms for participant screening which may introduce recall bias. Further, the change over time after the concussive event was beyond the scope of the current study. Future research that includes longitudinal testing of symptomatic and non-symptomatic post-concussed individuals starting within the first 3 weeks of injury may identify early indicators of late phase cervical spine deficits. These early indicators are key as patients often don’t engage with the health care system for more than a few weeks after concussion.

Another limitation of the study was that additional objective measurements to identify oculomotor, vestibular and cognitive impairments were not performed. Identification of impairments and classifying the respective subtypes of concussion would have enhanced this study.

The fact that we did one trial of joint position error (JPE), rather than multiple trials was another limitation of this study. We considered clinical pragmatism and participant’s time as a rationale for the trial of JPE. Individuals may have had improved JPE scores with repeated performance of the movements for JPE testing, which could result in a practice effect across both groups. Future research should consider repeated testing of JPE at multiple time points.

Lastly, while our methodology has some clear strengths, such as including definitive concussive screening questions recommend by expert consensus, our decision to include participants 18–50 years-old restricts minors from our sample limits the generalizability of our findings. Minors have a substantial risk for concussions due to greater relative participation in competitive sports and contact recreational activities. Previous research has found a high prevalence (86%) of impaired cervical spine joint mobility in children and adolescents receiving physical therapy treatments following a concussion [53]. Therefore, consideration of the inclusion of minors in future longitudinal studies may be helpful.

## Conclusion

The current study adds to existing evidence by sharing the extent of post-concussion cervicogenic impairments as revealed by clinical testing for people who did not actively seek rehabilitative care. The reader is cautioned about extending the results into youth populations as minors were not tested in this current work. Cervical spine screening for joint mobility and JPE is a reasonable clinical recommendation to detect important musculoskeletal impairments that could limit outcomes and patient wellness if left unresolved. Identifying and addressing these musculoskeletal impairments early in people with concussion may prove important in preventing the development of chronic cervical dysfunction and minimizing functional limitations and disability. The next steps in this line of inquiry could be initiating longitudinal testing that starts immediately after the injury that adds complimentary objective testing such as oculomotor or balance measures and additional cervical spine examination and injury history. The current work finds that individuals with a prior history of concussion experience sensorimotor and cervical spine joint mobility deficits that may often persist despite low concussive symptom scores. Although these impairments are not overtly noticed by the individual they are revealed with assessment of the cervical spine.

## Data Availability

The participants of this study did not give written consent for their data to be shared publicly, so due to the sensitive nature of the research supporting data is not available.
